# Association between smoking, and hospital readmission among inpatients with psychiatric illness at an academic inpatient psychiatric facility, 2000–2015

**DOI:** 10.1016/j.abrep.2019.100181

**Published:** 2019-04-09

**Authors:** Robert Kagabo, Jaewhan Kim, Jon-Kar Zubieta, Kristi Kleinschmit, Kolawole Okuyemi

**Affiliations:** a Department of Family & Preventive Medicine, University of Utah School of Medicine, 375 Chipeta Way Ste, A, Salt Lake City, UT 84108, United States of America; b Department of Health Promotion and Education, University of Utah College of Health, 250 S 1850 E, Salt Lake City, UT 84112, United States of America; c Department of Psychiatry, University of Utah School of Medicine, 501 Chipeta Way, Salt Lake City, UT 84108, United States of America

**Keywords:** Smoking, Psychiatric hospital readmission, Psychiatric illness

## Abstract

**Introduction:**

Smoking rates are up to 2–4 times higher among individuals with mental illness. Hospital readmissions for patients with psychiatric illness within a year of discharge are also high, and there is limited evidence of associations between smoking and these readmissions.

**Methods:**

This study was a secondary data analysis using clinical data of psychiatric inpatients with initial admissions between the years 2000 and 2015. Following a descriptive analysis, logistic regression models were fitted to explore relationships between smoking and psychiatric hospital readmission within 30 days and a year of discharge.

**Results:**

A total of 5439 patients with average age of 30.18 ± 15.97 were identified. Of this number, 47.0% were current smokers and 53.0% were never smokers. Within 30 days of discharge, 11% of the current smokers were readmitted compared to 9% of never smokers. The primary diagnoses with highest proportion of smokers were, opioid or substance use disorders (80.0%), schizophrenia (70.7%), alcohol dependence (68.2%), and bipolar disorders (50.8%). About 31% of current smokers were readmitted within one year of discharge compared to 26% of never smokers. Adjusted odds ratios for readmission within 1-year of discharge were, bipolar disorders (1.41, p = 0.01), schizophrenia (2.33, p < 0.001), and opioid/substance dependence (1.55, p = 0.01).

**Conclusion:**

Significant relationships exist between smoking and readmission for patients with psychiatric illness. Smokers are more likely to be readmitted within 30 days or one year after discharge. Interaction of smoking and certain specific diagnoses significantly increases readmission.

## Highlights

• Smoking rates are high among people with psychiatric illness.
• Significant associations exist between smoking and readmission for psychiatric patients.
• Smokers are more likely to be readmitted within 30 days or one year of discharge.
• Interaction of smoking and some psychiatric diagnoses increases readmission chances.

## 1 Background

Although individuals with mental illness make up only 22% of the total United States population, an estimated 44% of cigarettes sold in the United States are smoked by individuals with mental illness and substance use disorders (Kandel, Huang, & Davies, 2001; Lasser et al., 2000; Satcher, 2000). Studies have reported that there are high rates of smoking among psychiatric patients with specific diagnoses such as bipolar disorders compared to other psychiatric diagnoses (Gonzalez-Pinto et al., 1998; Grant, Hasin, Chou, Stinson, & Dawson, 2004). The complex relationship that exists between smoking and psychiatric illness increases tobacco-related risks of mortality and morbidity among individuals with psychiatric illness who smoke (Aubin, Rollema, Svensson, & Winterer, 2012). In some studies, cigarette smoking has been reported as a significant predictor of suicidal behavior among psychiatric patients (Breslau, Schultz, Johnson, Peterson, & Davis, 2005; Crockford, Kerfoot, & Currie, 2009). For example current smoking predicted suicidal thoughts, and suicidal attempts (odds ratio 1.82, 95% confidence interval 1.22–2.69) among young adults with psychiatric disorders (Breslau et al., 2005). On the other hand, incomplete remission from a psychiatric illness has been observed as a predictor of onset of daily smoking and later progressing to nicotine dependence (Breslau, Novak, & Kessler, 2004; Crockford et al., 2009). Individuals with psychiatric illness are also reported to be heavier smokers with very high nicotine dependence levels (Crockford et al., 2009; Lasser et al., 2000). Other researchers have suggested that there is an association between increased smoking rates and psychiatric diagnosis (Hughes, Hatsukami, Mitchell, & Dahlgren, 1986; Procyshyn, Ihsan, & Thompson, 2001). The objective of this study was to investigate the association between smoking and psychiatric hospital readmission.

Some history of smoking in psychiatry hospitals is critical to understand tobacco-related research among individuals with psychiatric illness. The hospitals in the United States banned tobacco use in 1993 but out of patient advocacy groups' outcries, an exception was permitted for inpatient psychiatry settings (Lawn & Pols, 2005; Prochaska, Hall, Delucchi, & Hall, 2014). Now more than half of inpatient psychiatry units allow smoking, and smoking cessation programs within these facilities are hardly available (Lawn & Pols, 2005; Prochaska et al., 2014; Prochaska, Gill, & Hall, 2004). Surgeon General Reports have been issued regarding smoking and the 1979 report recognized smoking as nicotine addiction while the 1988 report outlined that pharmacologic and behavioral processes of addiction for nicotine are similar to those of other drugs such as cocaine and heroin (Aubin et al., 2012). Using samples from the National Epidemiologic Survey on alcohol, investigators found that between 21% and 31% of individuals with a nicotine dependence also had some other psychiatric disorders or alcohol use disorder (Aubin et al., 2012; Grant et al., 2004). Brown et al. also found that the incidence of illicit drug use was higher among adolescents who smoked compared to those who did not smoke (Brown, Lewinsohn, Seeley, & Wagner, 1996).

Due to the complex relationship between psychiatric illness and smoking, both the American Psychiatric Association and the U.S. Public Health Service have recommended integrating smoking cessation with treatment of psychiatric illness (Dalack & Glassman, 1992; Hughes & Frances, 1995; Prochaska et al., 2004). However, individuals with psychiatric and substance use disorders are widely excluded in smoking cessation programs (Blanco et al., 2008; Blanco et al., 2008; Le Strat, Rehm, & Le Foll, 2011). While some research has been done regarding smoking among individuals with psychiatric illness, there is limited evidence of relationships between smoking, and hospital readmissions for inpatients with psychiatric illness. Readmissions for patients with psychiatric illness especially within the first year of their discharge are high and call for multiple interventions (Blader, 2004; Kagabo et al., 2016). Readmissions of patients with psychiatric illness that occur early following discharge are costly to patients' families, the healthcare system, and are a public health concern due to the disruptions in many of patients' functioning including social relationships (Habit, Johnson, & Edlund, 2018; Kagabo et al., 2016; Kolbasovsky, 2009). Smoking rates among individuals with psychiatric illness are also significantly high at a rate 2–4 times higher than in the general population (Aubin et al., 2012; Lising-Enriquez & George, 2009). Little is known about the relationship between cigarette smoking and psychiatric inpatient readmission. In this paper, we report the findings of a study that examined the association of smoking, and psychiatric hospital readmission at an academic inpatient psychiatry facility. The hypothesis for the study was that psychiatric inpatients who smoke are more likely to be readmitted for inpatient psychiatric care within 30 days or one year of discharge compared to psychiatric inpatients who do not smoke. Findings will inform healthcare providers and policy makers about the contribution of smoking to readmission rates and highlight the need to develop interventions to address smoking among psychiatric patients in hospital settings.

## 2 Methods

This study was a secondary data analysis using clinical data of psychiatric inpatients admitted to the University of Utah Neuropsychiatric Institute (UNI) between 2000 and 2015. Participants selected were those whose smoking status upon initial inpatient admission was indicated as either current smoker, or never smoker. The variables were not redefined by investigators so the intended clinical meaning was used. For example current smoker means patients who were current smokers, and never smoker means patients who had never smoked at the time of their hospital admissions. Clinical diagnoses such as schizophrenia, psychosis, and substance dependence were also not redefined. These diagnoses were used in the data analysis as originally recorded in the clinical data. Participants' demographic data including, age, race, gender, and smoking status were collected and analyzed. Participants were categorized in age groups as follows 4–11 children, 12–17 adolescents, 18–30 young adults, 31–64 middle age, and 65 or older for older adults. UNI uses these age groups to differentiate, children, adolescents, and adults. These age categorizations have also been used in many psychological studies with first appearance in Jean Piaget's work of stages of cognitive development (Esteve & Marquina-Aponte, 2012; Kagabo et al., 2017a; Lansford et al., 2008). In addition to smoking status, information on a number of psychiatric diagnoses such as, schizophrenia, bipolar disorders, substance dependence, psychosis, or posttraumatic stress disorders were obtained.

Descriptive statistics such as mean, percentages, and frequencies were calculated to describe the study sample, and to compare patients' characteristics by smoking status using chi square tests. In addition, bivariate and multivariate logistic regression models were fitted to obtain odds ratios and examine the associations between smoking and psychiatric hospital readmissions. Inpatient readmission was the dependent variable dichotomized as readmitted or, not readmitted. The independent variables included smoking status also dichotomized, sex, age group, race, and primary diagnosis for both readmissions within one year and within 30 days of discharge. We limited our interaction variables to readmissions within 30 days of discharge because the affordable care act (ACA) seeks to lower Medicare spending by limiting multiple readmissions in a short period of time (Kagabo et al., 2017b; McHugh & Ma, 2013). Readmissions in a short period of time are usually readmissions within 30 days of hospital discharge. (Kagabo et al., 2017b; McHugh & Ma, 2013).

Regression models were specified using the backward elimination method (Dunkler, Plischke, Leffondre, & Heinze, 2014). This method was beneficial in exploring other possible associations between smoking, and other independent variables. Readmission was the dependent variable, and smoking status was one of the independent variables. At p = 0.05, independent variables that were not significant were removed one at a time until the models that fit better were reached. In an effort to examine relationships between smoking and readmission, the specific primary diagnoses were individually controlled for in the multivariate logistic models as covariates. For readmissions within 30 days of discharge, interaction variables were created between smoking status and the primary diagnoses. Ethical approval for the study was obtained from the University of Utah Institutional Review Board.

## 3 Inclusion/exclusion

Participants were individuals who had received a psychiatric diagnosis with their initial inpatient admission at UNI between the years 2000 and 2015. All participants must have had at least a year of observation from their initial discharge to be included in the study. The one year period of observation was intended to give participants equal opportunity for the event of readmission within a year of initial discharge. After exclusion of participants whose smoking status was missing, there were 5777 unique participants who remained eligible and were categorized as either, current smokers, never smokers, or unknown smoking status. Smoking definition was based on clinical data as collected and categorized in the three mentioned categories at the time of initial inpatient admission. For a comparison of current smokers to never smokers, 338 participants out of 5777, whose smoking status was unknown were further excluded from the study and total number of participants left was 5439.

## 4 Results

### 4.1 Descriptive results

The final number of participants was 5439 of which 52.9% (n = 2881) were never smokers, and 47.0% (n = 2558) were current smokers. Among the never smokers, 25.8% (n = 742) were readmitted within one year of their initial discharge compared with 30.9% (n = 790) of the current smokers. The percentage of smokers was higher among males (54.3%) compared to females (39.7%) as shown in Table 1. The majority of the participants were from three major age groups, 12–17, 18–30, and 31–64 respectively identified as adolescents, young adults, and middle age. The highest percentage of smokers were observed among the young adults age group 18–30 among whom 62.7% of 1502 were current smokers, and the middle age group 31–64 years with 58.3% of 2136 being current smokers (Table 1). The majority of the participants were white (n = 4797) and 48.1% of whom were current smokers. Results also showed that of the 110 African American participants, 40.9% were current smokers and of the 35 Native Indian or Native Alaska participants 51.4% were current smokers (Table 1). The proportion of current smokers was highest among those with opioid use and substance dependence (80.0%), followed by those with schizophrenia (70.7%), alcohol dependence (68.2%), other psychotic disorders (51.7%), bipolar disorders (50.8%), and posttraumatic stress disorders (48.9%). The diagnoses with the least smoking rates were anxiety disorders (23.2%), mood disorders (31.3%), and depressive disorders (31.6%). The relationship between smoking and hospital readmission was statistically significant with chi square test results of (17.61, p < 0.001), see Table 1.

**Table 1:** Summary of smoking status by readmission, and other demographics (2000–2015).

Variables	Smoker				Total N = 5439	*χ*^2^	P
	Never		Current				
	n	(%)	n	(%)			
Readmission						17.61	0.000
Not readmitted	2139	54.75	1768	45.25	3907		
Readmitted	742	48.47	790	51.57	1532		
Sex						117.19	0.000
Male	1250	45.69	1486	54.31	2736		
Female	1631	60.34	1072	39.66	2703		
Age group (years)						800.57	0.000
4–11	210	97.67	5	2.33	215		
12–17	1115	77.92	316	22.08	1431		
18–30	560	37.28	942	62.72	1502		
31–64	891	41.71	1245	58.29	2136		
65+	105	67.74	50	32.26	155		
Race						25.56	0.001
Native Hawaiian/Pacific Island	9	45.00	11	55.00	20		
American Indian/Alaska N	17	48.57	18	51.43	35		
Asian	32	69.57	14	30.43	46		
Black or African American	65	59.09	45	40.91	110		
Other	170	62.04	104	37.96	274		
Refused/unknown	24	64.86	14	35.14	37		
White/Caucasian	2487	51.88	2307	48.12	4797		
Missing	77	62.60	46	37.40	123		
Primary diagnosis						652.61	0.000
Other	262	36.39	458	63.61	720		
Depressive disorders	1452	68.36	672	31.64	2124		
PTSD	24	51.06	23	48.94	47		
Bipolar disorders	233	49.16	241	50.84	474		
Schizophrenia	72	29.27	174	70.73	246		
Psychotic disorders	71	48.30	76	51.70	147		
Opioid/substance depend	40	20.00	160	80.00	200		
Anxiety disorders	73	76.84	22	23.16	95		
Mood disorders	397	68.69	181	31.31	578		
Alcohol dependence	257	31.81	551	68.19	808		
Never %, current %, total	2881	52.97	2558	47.03	5439		

### 4.2 Regression results

When examining readmission within 30 days of discharge the odds of readmission for current smokers was 1.28 (p = 0.01; 95% CI = 1.07, 1.52) for the unadjusted logistic regression model, but with a different outcome for the adjusted model where the odds ratio was 1.14 (p = 0.25; 95% CI = 0.91, 1.43) as seen in Table 3. Within one year of discharge, the odds of readmission for psychiatric patients who were current smokers was 1.29 (p < 0.001; 95% CI = 1.14, 1.45) in the unadjusted logistic regression model and 1.33 (p < 0.001; 95% CI = 1.16, 1.52) in the adjusted model (Table 2). Age group was not a significant factor for readmissions within 30 days of discharge but was found significant for readmissions within a year of discharge (Table 2 and Table 3). Logistic regression results for race as an independent variable both in the unadjusted and adjusted models did not reveal any significant associations between race and hospital readmissions. While there were no observed significant associations between gender and readmission within 30 days of discharge, results showed a significant association within a year of discharge. The odds ratio for readmission of females compared to males was 1.19 (p = 0.01; 95% CI = 1.05, 1.35).

**Table 2:** Association between psychiatric hospital readmission, smoking, and psychiatric diagnoses (2000–2015).

Variables	Bivariate/unadjusted			Multivariate/adjusted		
	Odds ratio	p-Value	95% CI	Odds ratio	p-Value	95% CI
Smoking						
Never						
Current	1.288	0.000	1.144–1.450	1.325	0.000	1.159–1.515
Sex						
Male						
Female	1.074	0.236	0.954–1.209	1.188	0.007	1.049–1.345
Age group						
4–11						
12–17	0.553	0.000	0.410–0.747	0.542	0.000	0.395–0.744
18–30	0.676	0.010	0.503–0.910	0.556	0.000	0.400–0.774
31–64	0.635	0.002	0.475–0.850	0.549	0.000	0.396–0.763
65+	0.623	0.038	0.399–0.973	0.587	0.026	0.368–0.937
Race						
Native Hawaiian/Pacific Is						
American Indian/Alaska N	1.565	0.476	0.458–5.352	1.797	0.357	0.517–6.246
Asian	1.182	0.758	0.357–3.918	1.338	0.638	0.398–4.501
Black or Afr. American	1.460	0.496	0.492–4.331	1.593	0.407	0.530–4.788
Other	1.443	0.491	0.509–4.096	1.673	0.339	0.582–4.807
Refused/unknown	0.469	0.283	0.118–1.869	0.527	0.368	0.130–2.128
White/Caucasian	1.182	0.747	0.429–3.259	1.381	0.538	0.495–3.855
Missing	0.481	0.206	0.155–1.496	0.595	0.375	0.189–1.873
Primary diagnosis						
Other	0.947	0.545	0.794–1.129	1.141	0.275	0.901–1.445
Depressive disorders	0.786	0.000	0.695–0.889	1.084	0.451	0.880–1.335
PTSD	0.975	0.938	0.513–1.852	1.140	0.700	0.586–2.217
Bipolar disorders	1.187	0.098	0.969–1.456	1.405	0.009	1.087–1.816
Schizophrenia	2.036	0.000	1.571–2.638	2.325	0.000	1.716–3.149
Psychotic disorders	1.498	0.020	1.066–2.107	1.772	0.003	1.212–2.591
Opioid/substance depend	1.360	0.043	1.009–1.831	1.551	0.011	1.106–2.176
Anxiety disorders	1.013	0.956	0.646–1.588	1.204	0.458	0.738–1.963
Mood disorders	1.174	0.093	0.974–1.415	1.336	0.035	1.021–1.748
Alcohol dependence	0.821	0.024	0.692–0.975	1.000	–	–

**Table 3:** Psychiatric hospital readmission within 30 days of discharge and smoking-diagnosis interaction (2000–2015).

Variables	Bivariate/unadjusted			Multivariate/adjusted		
	Odds ratio	p-Value	95% CI	Odds ratio	p-Value	95% CI
Smoking						
Never						
Current	1.276	0.007	1.067–1.524	1.141	0.252	0.911–1.429
Sex						
Male						
Female	1.087	0.358	0.910–1.299	1.185	0.073	0.984–1.425
Age group						
4–11						
12–17	0.714	0.141	0.456–1.118	0.748	0.226	0.467–1.197
18–30	0.860	0.505	0.553–1.339	0.788	0.339	0.484–1.284
31–64	0.776	0.252	0.503–1.198	0.784	0.327	0.482–1.276
65+	1.077	0.816	0.577–2.009	1.188	0.605	0.618–2.284
Primary diagnosis						
Other	1.048	0.723	0.809–1.358	1.986	0.032	1.063–3.711
Depressive disorders	0.769	0.006	0.638–0.928	1.622	0.105	0.904–2.911
PTSD	1.083	0.867	0.427–2.749	2.029	0.203	0.683–6.030
Bipolar disorders	1.160	0.333	0.859–1.567	1.342	0.445	0.631–2.857
Schizophrenia	2.322	0.000	1.671–3.225	3.819	0.002	1.658–8.796
Psychotic disorders	1.810	0.009	1.158–2.829	3.464	0.001	1.687–7.112
Opioid/substance depend	1.011	0.965	0.631–1.618	1.906	0.091	0.903–4.025
Anxiety disorders	0.951	0.886	0.476–1.900	1.847	0.175	0.760–4.486
Mood disorders	1.290	0.062	0.988–1.686	2.475	0.004	1.337–4.583
Alcohol dependence	0.626	0.002	0.469–0.836	1.00	–	–
Prim diagnosis ∗ smoking						
Bipolar disorders	1.697	0.004	1.181–2.437	2.186	0.020	1.338–4.216
Schizophrenia	2.470	0.000	1.692–3.605	1.152	0.714	0.541–2.450
Alcohol dependence	0.729	0.059	0.525–1.012	1.380	0.353	0.700–2.720

*Other interaction variables although had positive odds ratios were not reported due to insignificancy.

### 4.3 Diagnoses regression results

Results for readmission models within 30 days of discharge showed some significant associations between smoking and readmission for certain diagnoses in the multivariate analyses. The adjusted odds of readmission for patients with bipolar disorders were not significant, 1.34 (p = 0.45; 95% CI = 0.63, 2.86) however, the odds ratio increased and were significant with the interaction variable of smoking and bipolar disorders (smoking ∗ bipolar), 2.19 (p = 0.02; 95% CI = 1.34, 4.22). Associations for readmissions within 30 days of discharge and patients with schizophrenia were significant in multivariate logistic regression models with odds ratios, 3.82 (p = 0.002; 95% CI = 1.66, 8.80). The interaction variables of both schizophrenia, and psychotic disorders and smoking however did not show significant results (Table 3). Among patients with psychotic disorders, the odds ratio for readmission within 30 days of discharge changed from the unadjusted odds ratio of 1.81 (p = 0.01) to the significant adjusted odds ratio of 3.46, p = 0.001 (Table 3). The association between mood disorders and readmission within 30 days of discharge was also significant in the adjusted models where the odds ratio was 2.48 (p = 0.004; 95% CI = 1.34, 4.58) as shown in Table 3. Alcohol dependence or withdrawal was eliminated in the multivariate logistic regression models for collinearity reasons.

Significant results were observed for association between certain diagnoses and readmissions within a year of discharge. The multivariate logistic regression models showed odds of readmission for bipolar disorders as 1.41 (p = 0.01; 95% CI = 1.09, 1.82), and schizophrenia as 2.33 (p < 0.001; 95% CI = 1.72, 3.15). Other diagnoses with significant associations included psychotic disorders, 1.77 (p = 0.003; 95% CI = 1.21, 2.59), opioid or substance dependence, 1.55 (p = 0.01; 95% CI = 1.11, 2.18), and mood disorders, 1.34 (p = 0.04; 95% CI = 1.02, 1.75) after controlling for the rest of the diagnoses (Table 2).

## 5 Discussion

The purpose of this study was to explore the relationship between smoking and psychiatric hospital readmission for patients with psychiatric illness. The results showed that current smokers were more likely to be readmitted within 30 days and one year of discharge compared to never smokers (Table 2, Table 3). Researchers studying 30-day readmission of patients with inflammatory bowel disease also found smoking as one of the comorbidities associated with increased risk of readmissions within 30 days (Micic et al., 2017). Other studies in the general population observed that smoking was a risk factor for hospitalization and readmission with some research reporting that patients who received tobacco dependence treatment had lower odds of readmission at 30 days postdischarge (Cartmell et al., 2018; El Solh, Brewer, Okada, Bashir, & Gough, 2004).

With the odds ratio for readmission within a year of discharge at 1.33, psychiatric patients who were current smokers had 33% increased chance of readmission compared to the never smokers. While these observed differences may appear small, they represent 5.1% excess readmissions per year with additional associated costs. The observed difference continues to add to the growing evidence that tobacco use places a toll upon individuals with mental illness, accounting for 200,000 of the 443,000 tobacco-related annual deaths (Schroeder & Morris, 2010). The results of 54.3% of males as current smokers compared to 39.7% of females as current smokers are consistent with smoking in the general population where men compared to women are more likely to report that they are current smokers (Weinberger, Smith, Funk, Rabin, & Shuter, 2017). With the adjusted odds ratio of readmission within a year for females compared to males at 1.19, the females had a higher chance of 19% of being readmitted than males. Although the majority of the patients were White which is the general trend in the state of Utah, results also showed that 40.9% of African Americans and 51.4% of American Indian or Alaska Natives were current smokers. These results mirror other studies that have shown trends nation-wide that certain minority populations tend to have high rates of smoking (Marshall et al., 2017). Data from the 2010 National Health Interview Survey however showed that non-Hispanic African American smokers made quit attempts at a rate of 59.1% compared to non-Hispanic White smokers whose quit attempt rate was 50.7%. The quit attempts resulted in successful quitting at rates of 3.3% among the African Americans compared to 6.0% among the Whites (Keeler et al., 2017; Prevention CfDCa, 2011).

In this study, the highest percentage of current smokers were observed among patients with opioid or substance dependence followed by, schizophrenia, alcohol dependence, psychotic disorders, bipolar disorders, and posttraumatic stress disorders. While there is a body of research regarding smoking and mental illness, the directions or distribution of associations of smoking among the different psychiatric disorders in the literature is not consistent. The common reports are of correlations between smoking and psychological problems such as depression and anxiety (Davoudi, Omidi, Sehat, & Sepehrmanesh, 2017; Fluharty, Taylor, Grabski, & Munafo, 2017). Studies have however observed smoking rates to be high in patients with schizophrenia and bipolar disorders (Kotov, Guey, Bromet, & Schwartz, 2010; Lasser et al., 2000; Lawrence, Hancock, & Kisely, 2013). This study specifies the different percentages of smokers in the different diagnoses with highest proportion seen among patients with opioid and substance dependence. These findings are consistent with other studies reporting higher associations of smoking and opiate drug use (Guydish et al., 2016). The results show that there is a significant relationship between specific psychiatry primary diagnoses and smoking, p < 0.001 (Table 1).

## 6 Strengths and limitations

One limitation was that the study was a secondary data analysis with clinical data not specifically collected to study relationships between smoking and readmission. Many participants were excluded because their smoking status was missing. It is possible that smoking status for these patients was missing because clinical data were not collected for the purpose of smoking studies. The data were also from a single academic psychiatric facility, which is a challenge to follow up on patients who may have been readmitted in a different facility. A strength for the study was the comparison of smoking within the different psychiatry diagnoses.

## 7 Conclusion

The largest proportion of smokers were observed in patients with the primary psychiatric diagnoses of, opioid or substance dependence followed by schizophrenia, alcohol dependence, psychotic disorders, and bipolar disorders. The results of this study show that when smoking interacts with certain specific psychiatric diagnoses such as bipolar disorders, the likelihood of psychiatric hospital readmission significantly increases. The study results also indicate a significant relationship between smoking and psychiatric hospital readmission that deserves further investigation. These findings may also imply that targeting smoking cessation interventions to patients with specific diagnoses like bipolar disorders may contribute to lowering readmission rates and improving the general health of patients with psychiatric illness. Psychiatry providers in inpatient psychiatric facilities may also benefit from forming partnerships with smoking cessation providers and integrating smoking cessation in inpatient psychiatric care. Additional research in the association between readmission and change in smoking status between initial admission and readmission may also be beneficial in establishing the relationship of smoking and readmission.

## Declarations of interest

None.

## Role of funding source

This work did not receive any specific grant from funding agencies in the public, commercial, or not-for-profit sectors.

## Contributors

Dr. Kagabo, the corresponding author wrote the first draft, performed data analysis and was the main contributor to the protocol design. Dr. Kagabo was supervised by Dr. Okuyemi. All the other authors contributed to the design and proof reading of the manuscript.

## Declaration of conflict of interest

All authors declare no conflict of interest.
